# Tree functional types simplify forest carbon stock estimates induced by carbon concentration variations among species in a subtropical area

**DOI:** 10.1038/s41598-017-05306-z

**Published:** 2017-07-10

**Authors:** Huili Wu, Wenhua Xiang, Xi Fang, Pifeng Lei, Shuai Ouyang, Xiangwen Deng

**Affiliations:** 1 Faculty of Life Science and Technology, Central South University of Forestry and Technology, Changsha, Hunan 410004 China; 2 Huitong National Station for Scientific Observation and Research of Chinese Fir Plantation Ecosystems in Hunan Province, Huitong, Hunan 438107 China

## Abstract

Forests contain one of the world’s largest carbon (C) pools and represent opportunities for cost-effective climate change mitigation through programmes such as the United Nations-led “Reducing Emissions from Deforestation and Forest Degradation” Programme (REDD). Generic estimates for the conversion of forest biomass into C stock are not sufficiently accurate for assessing the utility of harvesting forest to offset carbon dioxide emissions, currently under consideration by the REDD Programme. We examined the variation in C concentration among tree species and tree functional types (classified based on leaf morphological and phenological traits) in a subtropical forest and evaluated the effects of these variations on stand-level estimations of C stock. This study was conducted in the Paiyashan Forest State Farm and the Dashanchong Forest Park, Hunan Province, China. C concentrations differed significantly among tree species (*P* < 0.0001) and were significantly higher in gymnosperm than angiosperm species. Estimations of stand C stocks were similar using either functional types or species- and tissue-specific C concentrations. The use of functional type classification to estimate stand C stock is an effective tool for implementing C sequestration trade and C credit programmes and the UN-REDD Programme in subtropical forests.

## Introduction

Forests are essential to the approximately 1.6 billion people who depend on them for food, water, fuel, medicines, tradition and livelihood^1^. Forests accumulate over 45% of the carbon (C) in the terrestrial biosphere and account for nearly half of terrestrial net primary production^2^. Therefore, forests play a vital role in regulating atmospheric C concentrations and our climate by natural C sequestration. Yet, on average, thirteen million hectares of forests disappear annually, often devastating the well-being of local communities^1^. Deforestation and forest degradation account for over 10% of greenhouse gas emissions^3^, making the reduction of emissions from deforestation and the increase of forest restoration important consideration for limiting global warming to +2 °C^4^. The United Nations Collaborative Programme on “Reducing Emissions from Deforestation and Forest Degradation in Developing Countries” (UN-REDD Programme) was launched to reduce forest emissions and enhance C stocks in forests while contributing to national sustainable development^1, 5^. As part of UN-based forest C accounting protocols^6, 7^, accurate measures of C concentrations for diverse tree species are needed to estimate the size of C stock in forest biomass. Studies show that C concentrations vary among the tree species^8–10^. Thus, one of the main challenges for implementing the UN-REDD Programme will be accounting for the effects of these variations on forest C stock estimates.

Forest C stocks are commonly estimated by multiplying forest biomass (dry weight) with a constant conversion factor (C concentration)^6, 7^. One widely accepted value for this conversion factor is 50.0% by mass^11, 12^, though an increasing number of studies indicate that C concentration varies significantly among tree species and tissues^8, 13^. In one instance, the C concentrations of 5 tree species common in subtropical forests varied from 47.9% to 57.7%^14^. The average C concentrations for tree tissues in 10 Chinese temperate species were 49.9% for stems, 50.3% for old branches, 50.8% for new branches, 51.4% for leaves, 47.1% for fine roots and 48.8% for coarse roots^8^. Ignoring species- and tissue-specific variations in C concentration and using the generic value of 50% could introduce between −6.2% to 7.0% error in stand C stock estimates from forest inventory data, or a 10% error in stand biomass C stock estimates from 32 neotropical tree species^8, 10, 13, 15, 16^. It is recognised that species- and tissue-specific variations in C concentration need be considered to meet the accuracy required by C sequestration trade, C credits^9^ and the UN-REDD Programme.

Even though species-specific C concentrations are preferred when estimating stand C stock, it is impractical to obtain the data for every species in highly diverse forest ecosystems. Generalizing C concentration based on tree phylogeny (e.g. gymnosperm and angiosperm species) and functional traits may provide a realistic alternative. This possibility is supported by research showing that C concentrations vary between functional types^10, 17, 18^. For example, the C concentration of woody tissue is greater in gymnosperm species (50.8%) than angiosperm species (47.7%)^17^, likely attributable to differences in lignin chemistry^19^. Gymnosperm species have more highly lignified stem wood^20^, while angiosperm species have higher concentrations of non-structural carbohydrates^21^, demonstrating how broad phylogenetic differences may inform measurements of C concentration. The relationships between functional traits and C concentrations and functional types and variations in C concentrations among tree species have not been fully examined.

Plant functional traits and their associated trade-offs control a variety of terrestrial ecosystem processes – including forest C stock, a key component of the global C cycle^22–24^. Leaves assimilate C through photosynthesis and stems accumulate the greatest C stock. The relevant functional traits to C concentration in leaves^22, 25^ include: leaf area (LA), specific leaf area (SLA), photosynthetic capacity, leaf nitrogen and phosphorus concentrations, dark respiration rate and leaf lifespan^22, 25^. For stems, the functional traits of note are maximum plant height, wood density (WD), wood specific gravity, relative growth rate (RGR) and mean annual increment of biomass (MAI)^8, 24^. WD has proven to be inconsistently correlated with C concentration^8, 10, 13, 15^. For instance, Elias and Potvin found that C concentration was highly positively correlated with WD^13^, while Thomas and Malczewski report a non-significant negative correlation with WD for gymnosperm and a reverse trend for angiosperms^10^. Most researches have shown no relationship between C concentration and WD^8, 15, 26^. Zhang *et al*. found a significant negative relationship between C concentration and MAI^8^. Martin and Thomas found that C concentration was not correlated with RGR^15^. SLA is frequently used in growth analysis because it is positively correlated to RGR across species, and tends to scale negatively with C shunting to more recalcitrant C-containing secondary compounds, such as tannins or lignin^27^. LA is negatively related to WD^28–31^, but no relationship between C concentration and LA has been reported. We investigated the correlation between five functional traits (WD, MAI, RGR, SLA and LA) and C concentrations, while controlling for phylogeny, to test their predictive value in subtropical tree species.

Few surveys of C concentration in subtropical tree species have been conducted to date. Zheng *et al*. examined differences in C allocation in forests under different management practices^14^ and Zhuo *et al*. developed compatible C content models of individual trees in a Chinese fir plantation^32^. The extent of variation in C concentration among subtropical tree species has not yet been addressed in subtropical forest. Thus, we examined species- and tissues-specific variations in C concentrations in eight common tree species among two functional types: two gymnosperm species (*Cunninghamia lanceolata* and *Pinus massoniana*) and six angiosperm species (*Alniphyllum fortunei*, *Choerospondias axillaris*, *Liquidambar formosana*, *Cyclobalanopsis glauca*, *Litsea rotundifolia* and *Schima superba*). The objectives of this study were to: (1) examine variations in C concentrations among tree species; (2) determine whether functional type classifications, based on phylogeny and functional traits, correspond with variations in C concentrations among tree species; and (3) compare estimates of stand C stocks using species- and tissues-specific, functional type and generic C concentrations.

## Materials and Methods

### Site description

The study was conducted at the Paiyashan Forest State Farm (latitude 26°24′–26°35′N, longitude 109°27′–109°38′E) in Jingzhou County, Hunan Province, China. The altitude of the farm ranges from 330 m to 1075 m above mean sea level. The farm is in the humid mid-subtropical monsoon climatic zone. The average annual temperature is 16.7 °C and the mean annual precipitation is 1250 mm. The parent material is purple sand shale. At altitudes below 600 m, the soil type is red soil, and at altitudes above 600 m, the soil type is characterised as yellow soil. These soils are classified as Alliti-Udic Ferrosols in the Chinese Soil Taxonomy, which corresponds to Acrisol in the World Reference Base for Soil Resources^33, 34^. The farm encompasses *C. lanceolata* plantations and several secondary forests (e.g. coniferous and deciduous mixed, deciduous and evergreen broadleaved mixed, and evergreen broadleaved forests) dominated by many native tree species^35^.

### Tree sampling and tissue sample preparation

According to leaf morphological and phenological traits^36^ and species composition, we sited eight forest types in Paiyashan Forest Farm. A 30 m × 30 m plot was established for each forest type. We selected 8 tree species in the eight forests which divided into two functional types, i.e., two gymnosperm species (*C. lanceolata* and *P. massoniana*) and six angiosperm species (*A. fortunei*, *C. axillaris*, *L. formosana*, *C. glauca*, *L. rotundifolia* and *S. superba*). Ten trees from each species were selected for C concentration sampling (except for 18 trees for *C. lanceolata*). Trees were selected to range in diameter at breast height (DBH) between the minimum and maximum observed values for a given species and along an even distribution. A total of 88 sample trees with DBH up to 0.51 m and height up to 30.2 m were harvested for C concentration measurements. Stand characteristics of the eight secondary forests, and tree species selected for C concentration measurement, are presented in Table S1.

All living branches were cut from canopy heights (upper, middle and lower) as close to the stem as possible and were immediately weighed. Three representative branches from each canopy height were selected, and all leaves were separated from every branch. Three fresh branch samples, weighing approximately 500–1000 g, were randomly sampled from three larger branches together with 3 corresponding leaf samples. A 5-cm thick disc was taken at 0–1.3 m, 1.3 m to half of total height and over half of total height of stem. Three wood discs were sampled from bark and stem without bark from each tree. A quarter sector of each wood disc was sampled for measuring C concentration and the remaining three-quarters was sampled for RGR and WD measurements. Roots were divided into coarse (diameter >2 mm) and fine roots (diameter <2 mm)^37^ and three samples were randomly collected from each. All samples were taken in October 2014 before leaf fall and transported to the laboratory for chemical analysis.

### Measuring C concentration

All samples were oven-dried to a constant mass at 70 °C. The samples were ground to a fine powder using a ball mill (0.25 mm), and 20–30 mg of powder for each sample was used to determine C concentration. The C concentration was measured by the oil-bath K_2_Cr_2_O_7_–H_2_SO_4_ titration method^14, 38^, and expressed as % dry mass.

### Measuring functional traits

We focused on two functional traits of leaves and three of stems because these traits are directly relevant to C sequestration, growth, survival and reproduction of trees^39–42^. LA (cm^2^) has important consequences for leaf energy and water balance^22^ and SLA (cm^2^ g^−1^) is an indicator of a trade-off in leaves between C gain and leaf longevity^22^. For stems, MAI (kg year^−1^) is the increase in biomass at the start of a given time interval. RGR (g kg^−1^ year^−1^) is a prominent indicator of plant strategy concerning productivity with environmental stress and disturbance regimes^27^. WD (g cm^−3^) captures trade-off in stems between growth and strength^23^. The data of these functional traits are easily obtained and are potentially correlated with C concentrations^8, 22–24, 43^.

To estimate LA and SLA, 15 intact and full grown leaves were randomly sampled at each canopy position (upper, middle and lower) from every tree. Leaves were then pooled to determine LA and weight for SLA. Each leaf length and width was measured to calculate LA. All leaves were washed with distilled water and oven-dried to a constant mass at 60 °C to measure the dry biomass for SLA.

A stem disk at breast height (1.3 m) from each tree sample was sanded and a digital scan was made with a scanner. Annual increments of DBH in the last 5 years were calculated on four directions (N, S, E and W) and averaged for each sampled tree. The values of MAI and RGR were calculated from annual incremental growth in tree biomass using allometric equations developed by Xiang^35^, as follows^8, 44^:

1 $$MAI=({M}_{2}-{M}_{1})/({t}_{2}-{t}_{1})$$

2 $$RGR=({\rm{In}}{M}_{2}-{\rm{In}}{M}_{1})/({t}_{2}-{t}_{1})$$

where *M*_1_ and *M*_2_ represent tree biomass at times *t*_1_ and *t*_2_, respectively.

WD was calculated as dry mass divided by field-moist wood volume for each sampled tree. A disk about 3–5 cm thick was sawed from the trunk at ~1.3 m height. The field-moist wood volume was measured by water-displacement. Wood samples were oven-dried at 100–110 °C to constant weight to obtain the dry mass.

### Phylogenetic analyses of functional traits

Traits that evolve slowly are considered subject to phylogenetic “constraint” and, thus, have a phylogenetic signal. The phylogenetic signal in C concentrations of stem, bark, branch, leaf, coarse root and fine root as well as functional traits (i.e. WD, LA, SLA, MAI and RGR), was quantified using the *K* statistic^45^ performed in the “picante” package^46^ in R^47^. The *K* statistic compares a trait distribution from a phylogenetic tree to a distribution expected under a Brownian motion model of evolution that represents a continuous evolutionary change and random distribution across the phylogenetic tree. The *K* value was calculated by the following formula:

3 $$K={\rm{observed}}({{\rm{MSE}}}_{0}/{\rm{MSE}})/{\rm{expected}}({{\rm{MSE}}}_{0}/{\rm{MSE}})$$

where the MSE_0_ is the mean squared error of the tip data calculated by the phylogenetically correct mean (MSE_0_) and the MSE is the mean squared error of the data measured by the variance-covariance matrix derived from the candidate tree^45^. A *K* = 1 implies that the observed trait distribution matches the Brownian motion model, while *K* < 1 implies more randomly distribution than a Brownian motion model and *K* > 1 implies higher phylogenetic signal or more conservatism than a Brownian motion model (i.e. trait similarity of related taxa)^15, 45, 48^. Statistical significance was tested by random permutation of traits across the tips of the phylogeny (n = 999). Traits were deemed significantly conserved if the observed *K* was in the upper 2.5% of the randomised *K* distributions. It should be noted that this null model of randomised *K* distributions corresponds to no phylogenetic signal, with K_null_ << 1^48^. Phylogenetic trees were created with Phylomatic (v3) based on the Angiosperm Phylogeny Group (APG) III system^49^.

Estimates of forest stand C stock. We used the measurements from four forests in Dashanchong Forest Park^50^ (28°23′–28°24′N, 113°17′–13°19′E), Changsha County, Hunan Province, China, to quantify the error in stand C stock estimated using the generic C concentration constant (50.0%) and the C concentrations measured in this study. A 1-ha permanent plot was established for each forest and, within each, 20 m × 30 m subplots were established. There were seven subplots for *C. lanceolata* plantation (CLF), 15 for coniferous mixed forest (PMF), 16 for deciduous mixed forest (CAF) and 14 for evergreen broadleaved forest (CGF)^51, 52^.

The inventory of tree species, according to percentage of biomass, for all four forests was: (1) 97% *C. lanceolata* biomass and 3% other deciduous and evergreen angiosperm biomass in CLF; (2) 49% *P. massoniana* biomass, 7% *C. glauca* biomass and 44% other deciduous and evergreen angiosperm biomass in PMF; (3) 58% *C. axillaris* biomass, 2% *L. rotundifolia* biomass and 40% other deciduous angiosperm biomass in CAF; and (4) 14% *C. glauca* biomass, 13% *C. axillaris* biomass and 73% other evergreen broadleaved biomass in CGF. C stocks (t C ha^−1^) were estimated using the generic C concentration, C concentrations of tree species and tissues, and C concentrations of average value of all tree species in specific functional type measured, given as CS_g_, CS_m_ and CS_f_, respectively.

### Data analysis

Bartlett and Levene tests were performed to determine if the data satisfied homogeneity of variance. Analysis of variance (ANOVA) and Tukey’s honest significant difference (HSD) test were used to detect significant differences between tree species and tissues. One-way ANOVA was used to compare differences of stand C stock estimates between CS_g_, CS_m_ and CS_f_. The relationships between C concentration and functional traits (LA, SLA, MAI, RGR and WD) were analysed using stepwise regression. Functional traits were selected as having significant effects on C concentrations according to the lowest Akaike information criterion (AIC). Phylogenetically independent contrasts (PIC) were tested using the “ape” package^53^ to determine the effect of phylogeny on the relationship between C concentrations and functional traits. All statistical analyses were performed in R^47^.

## Results

### Species- and tissue-specific variations in C concentration

**Figure 1:**
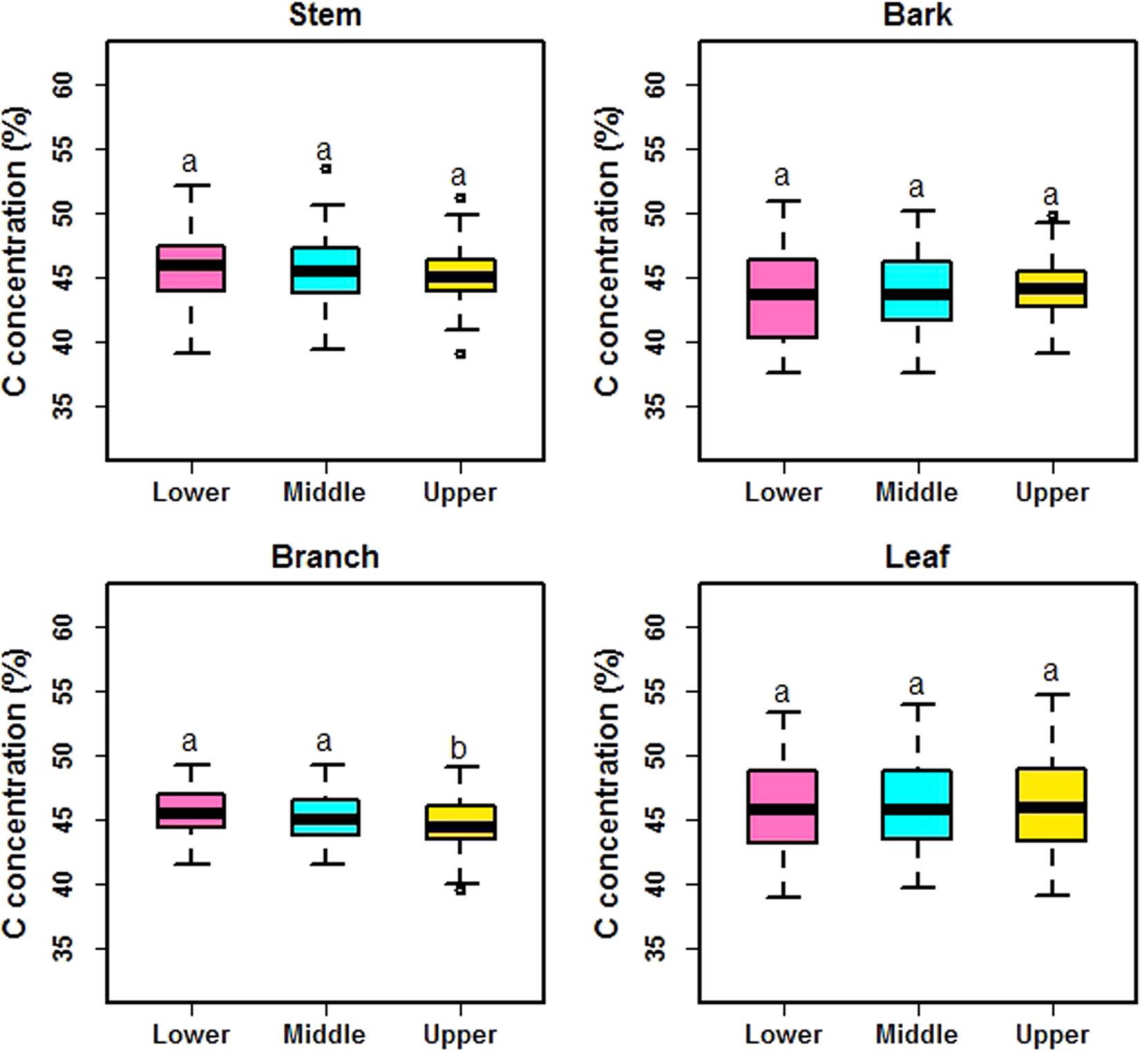
Effects of position on C concentrations in stems, bark, branches and leaves across all tree species. Lower, middle and upper for stem and bark refer to 0–1.3 m height, 1.3 m to half of total height and above half of total height of stem, respectively. Lower, middle and upper for branch and leaf refers to the position within the canopy. For the boxplots: circles are outliers; vertical bars are data ranges defined as 1.5 × the inter-quartile range; horizontal lines within the boxes are median values; and the upper and lower bounds of the boxes are the third and first quartiles, respectively. Letters indicate significant difference at *P* < 0.05 (n = 88).

Significant effects of species, tissues and their interaction on C concentrations were observed (Table S2). Tree species, tissues and their interactive effect accounted for 35.41%, 17.89% and 18.97% of the total variance in C concentrations, respectively. C concentrations in other tissues did not significantly differ among the canopy or stem positions (Fig. 1), except among branches (*F*_2,236_ = 4.154, *P* = 0.017).

C concentrations differed significantly among tree species (*P* < 0.0001). Average C concentration was highest in *C. lanceolata* (47.9 ± 2.5%, mean ± SD) and lowest in *A. fortunei* (42.3 ± 1.0%) (Table 1). Overall, the order of tree species based on highest to lowest C concentrations was as follows: *C. lanceolata* > *P. massoniana* > *L. rotundifolia* > *C. axillaris* > *S. superba* > *L. formosana* > *C. glauca* > *A. fortunei*. C concentrations for a specific tissue also varied among tree species. The highest C concentrations were found for stems in *P. massoniana*, branches in *L. rotundifolia* and bark, leaves, coarse roots and fine roots in *C. lanceolata* among the eight tree species, whereas the lowest C concentrations were found for stems and coarse roots in *C. glauca*, bark in *A. fortunei*, branches in *S. superba*, leaves in *L. formosana* and fine roots in *L. rotundifolia* (Table 1).

**Table 1:** Average C concentrations in each tissue of eight subtropical tree species.

Tree species	C concentration (%)						
	Stem	Bark	Branch	Leaf	Coarse root	Fine root	All tissues
**Gymnosperm species**							
*C. lanceolata*	47.5 (1.2)	49.6 (2.9)	45.6 (0.8)	49.8 (1.7)	48.0 (1.5)	46.9 (3.4)	47.9 (2.5)
*P. massoniana*	49.1 (1.2)	46.7 (1.4)	47.2 (1.2)	47.2 (2.2)	46.6 (1.7)	42.4 (1.5)	47.4 (0.9)
*All gymnosperm species*	48.1 (1.4)	48.5 (2.8)	46.1 (1.2)	48.8 (2.2)	47.5 (1.7)	45.4 (3.6)	47.4 (2.6)
**Angiosperm species**							
*A. fortunei*	44.0 (0.8)	40.8 (1.6)	43.3 (1.2)	42.9 (1.8)	41.7 (2.2)	39.1 (3.1)	42.3 (1.0)
*C. axillaris*	44.7 (1.1)	44.8 (1.3)	45.0 (0.8)	44.2 (2.4)	43.3 (2.0)	40.2 (2.5)	44.3 (0.5)
*L. formosana*	46.8 (0.9)	41.7 (1.1)	44.5 (0.7)	41.8 (1.2)	42.3 (1.8)	39.1 (1.3)	43.7 (0.5)
*C. glauca*	43.5 (1.5)	41.8 (1.2)	44.0 (0.5)	44.0 (1.7)	41.0 (1.8)	42.3 (2.7)	42.6 (0.8)
*L. rotundifolia*	46.1 (0.9)	46.4 (1.6)	47.3 (1.0)	46.8 (1.6)	43.0 (1.5)	36.6 (2.8)	46.1 (0.7)
*S. superba*	44.2 (1.1)	44.0 (2.2)	43.0 (0.9)	46.9 (1.4)	42.5 (2.8)	42.3 (3.1)	43.9 (1.1)
*All angiosperm species*	44.9 (1.6)	43.3(2.5)	44.5 (1.7)	44.5 (2.5)	42.3 (2.1)	40.0 (3.3)	43.8 (2.4)
All species	45.9 (2.1)	45.0 (3.6)	45.1 (1.7)	45.9 (3.2)	44.0 (3.2)	42.0 (4.3)	44.7 (3.3)

Standard deviations from the mean are provided in parentheses. Sample size is equal to ten for all species except *C. lanceolata* where n = 18.

For a given tree species, C concentrations significantly differed among tissues (*P* < 0.0001). From highest to lowest, the C concentration of tissues for each tree species ranked accordingly: leaf > bark > coarse roots > stem > fine root > branch for *C. lanceolata*; stem > branch = leaf > bark > coarse root > fine root for *P. massoniana*; stem > branch > leaf > coarse root > bark > fine root for *A. fortunei*; stem > branch > coarse root > leaf > bark > fine root for *L. formosana*; branch > bark > stem > leaf > coarse root > fine root for *C. axillaris*; branch > leaf > bark > stem > coarse root > fine root for *L. rotundifolia*; branch = leaf > stem > fine root > bark > coarse root for *C. glauca*; and leaf > stem > bark > branch > coarse root > fine root for *S. superba* (Table 1).

### Phylogenetic distribution of C concentration and functional traits

**Figure 2:**
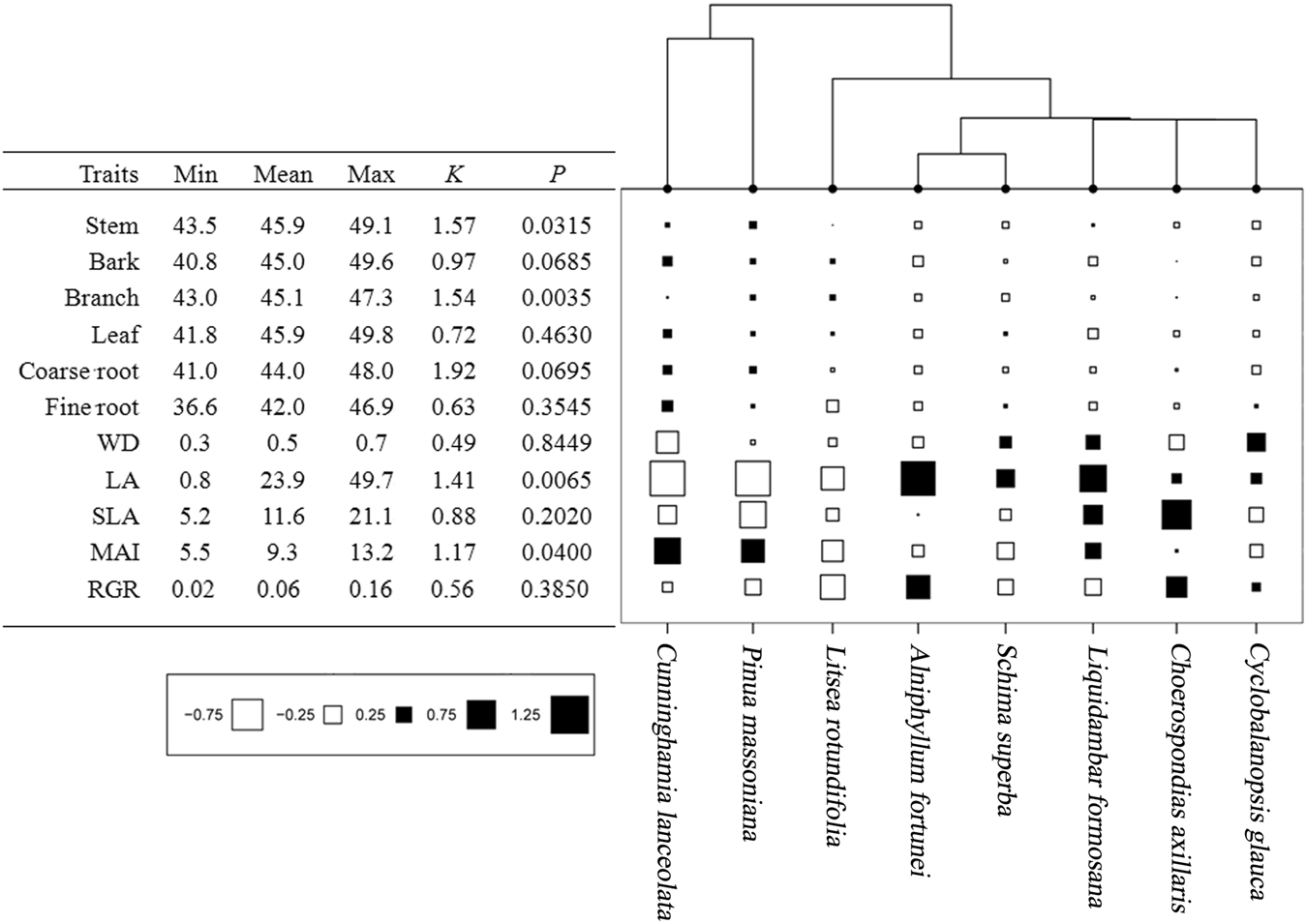
Phylogenetic conservation of functional traits. Symbol size indicates the percent of functional trait values greater (solid black) or less (solid white) than the mean values for each tree species. The adjoining table provides the C concentration range for each tissue and *K* statistic with p-value. *K* statistics < 1 indicate more randomly distribution, while *K* statistics > 1 indicate a higher conservation of the traits than expected by random Brownian distribution. *P* values refer to the results comparing the observed *K* to a null distribution of *K* values obtained by shuffling the traits across the tips of the phylogeny 999 times. The *P* values were calculated by dividing the number of all null *K* values greater than the observed *K* by 999.

The phylogenetic tree of tree species clustered into two distinct clades: one clade for the two gymnosperm species and another for the six angiosperm species, supporting the classification of trees into two broad functional types. *K* statistics for the C concentrations of stem (*K* = 1.57), branch (*K* = 1.54), coarse root (*K* = 1.92), LA (*K* = 1.41) and MAI (*K* = 1.17) indicated the phylogenetic conservation of these traits (Fig. 2). *K* statistics for the C concentrations of bark (*K* = 0.97), leaf (*K* = 0.72), fine root (*K* = 0.63), WD (*K* = 0.49), SLA (*K* = 0.88) and RGR (*K* = 0.56) indicated a random distribution (Fig. 2).

### C concentrations and functional types

**Figure 3:**
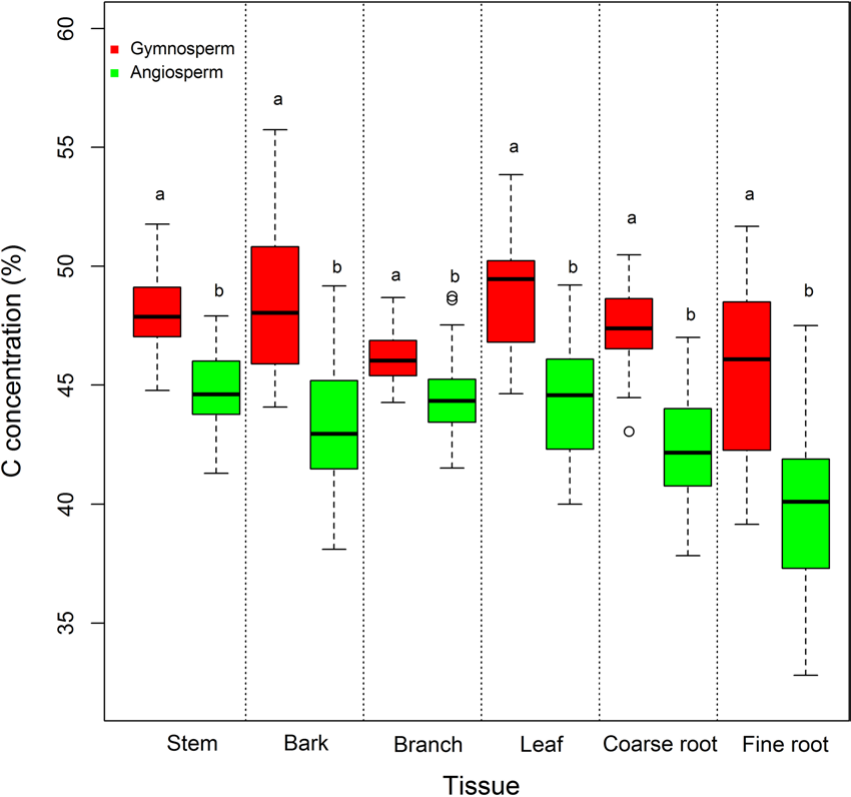
Comparison of C concentrations in stems, bark, branches, leaves, coarse roots and fine roots among functional types. For the boxplots: circles are outliers; vertical bars are data ranges defined as 1.5 × the inter-quartile range; horizontal lines within the boxes are median values; and the upper and lower bounds of the boxes are the third and first quartiles, respectively. Letters indicate significant difference at *P* < 0.0001 (gymnosperm, n = 28; angiosperm, n = 60).

C concentrations in gymnosperm species (47.4 ± 2.6%) were significantly higher than those in angiosperm species (43.8 ± 2.4%) across all tissues (*P* < 0.0001; Fig. 3). C concentrations in gymnosperm species were greatest in leaf (48.8 ± 2.2%), while the highest concentrations occurred in stem (44.9 ± 1.6%) for angiosperm species. Fine root contained the lowest C concentration in both gymnosperm (45.4 ± 3.6%) and angiosperm (40.0 ± 3.3%) species.

### C concentrations and functional traits

**Figure 4:**
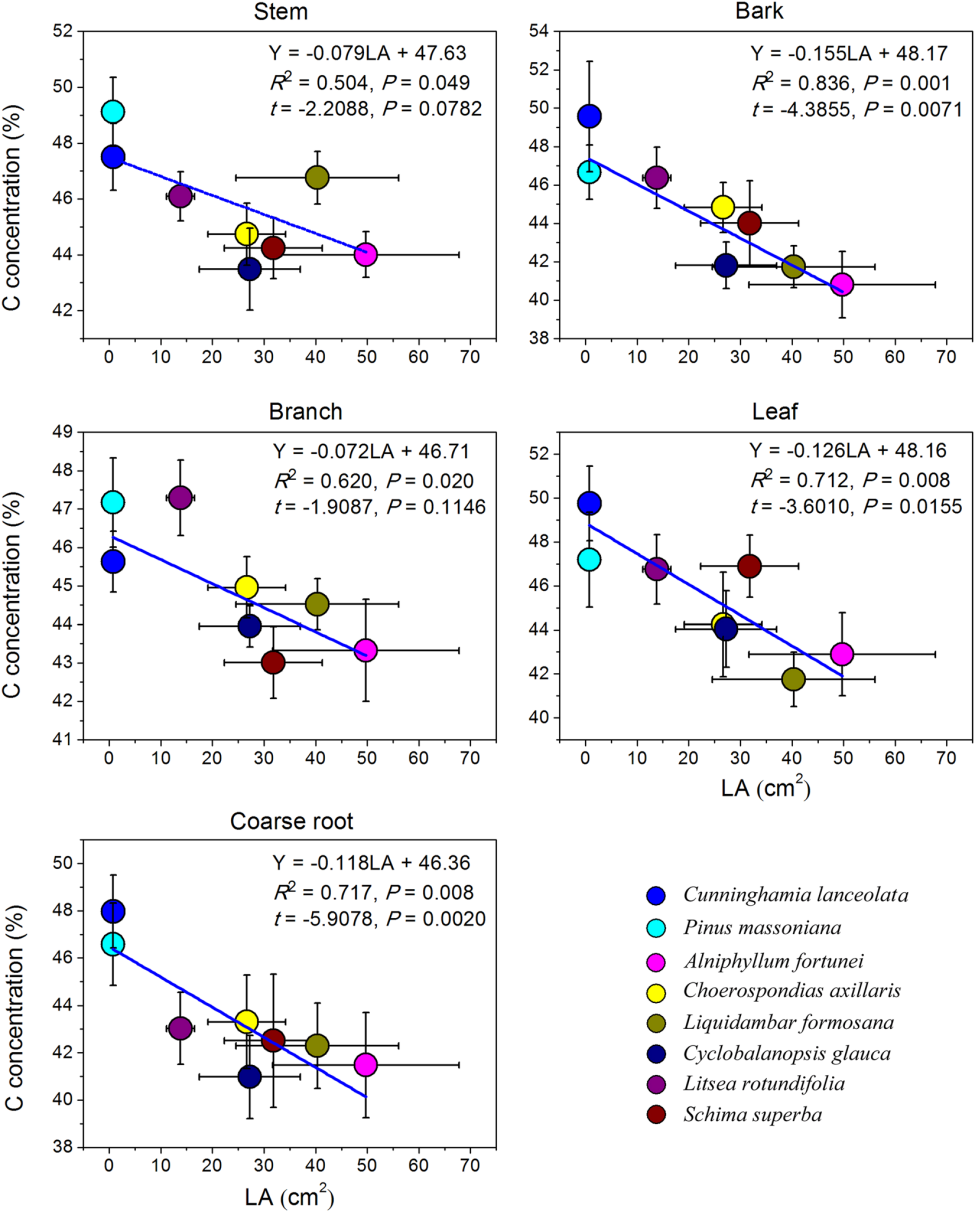
Correlation between average C concentrations in stems, bark, branches, leaves and coarse roots with leaf area. The test statistic (*t*) and p-value (*P*) were calculated by Phylogenetically Independent Contrasts.

Significant negative relationships were observed between LA and average C concentrations of stems (*F*_1,6_ = 6.089, *P* = 0.049), bark (*F*_1,6_ = 30.53, *P* = 0.001), branches (*F*_1,6_ = 9.803, *P* = 0.020), leaves (*F*_1,6_ = 14.860, *P* = 0.008) and coarse roots (*F*_1,6_ = 15.19, *P* = 0.008) across all eight tree species (Fig. 4). For all sampling angiosperms trees, C concentrations were negatively correlated with LA in stems (*F*_1,48_ = 4.638, *P* = 0.036), bark (*F*_1,48_ = 21.200, *P* < 0.0001), branches (*F*_1,48_ = 22.090, *P* < 0.0001), leaves (*F*_1,48_ = 10.780, *P* = 0.002) and roots (*F*_1,48_ = 3.027, *P* = 0.088) (Fig. S1). Significant or marginal significant negative correlations between C concentrations of each tissue (except branch) and LA were apparent when testing for the influence of PIC (Fig. 4). There was no significant relationship between LA and C concentrations in fine roots (*F*_1,6_ = 2.145, *P* = 0.193). There were no significant relationships between the average C concentrations of the eight tree species and any other functional traits: SLA, MAI, RGR and WD (Table S3).

### Error in estimation of forest C stock

**Figure 5:**
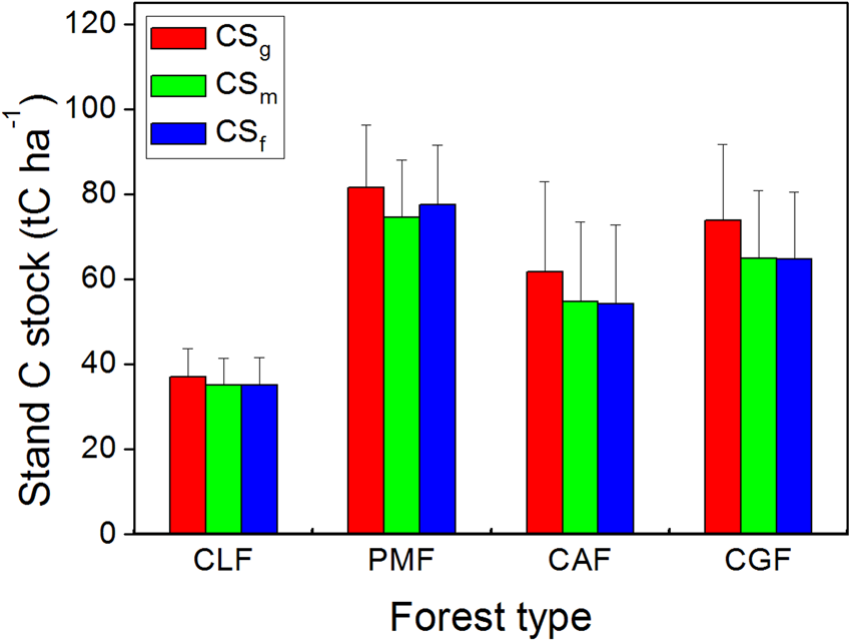
C stock estimates in four forest stands using generic C concentration, C concentrations of tree species and tissues or C concentrations of functional types. The CS_g_, CS_m_ and CS_f_ refer to the C stocks estimated by using generic C concentration, C concentrations of tree species and tissues, and C concentrations of average value of all tree species in specific functional types, respectively. The CLF, PMF, CAF and CGF represent *C. lanceolata* plantation (n = 7), coniferous mixed forest (n = 15), deciduous mixed forest (n = 16) and evergreen broadleaved forest (n = 14), respectively.

Stand C stocks estimated using generic C concentration (CS_g_) and C concentration of species and tissue (CS_m_) and functional type (CS_f_) were 37.00, 35.09 and 35.15 t C ha^−1^ for the CLF plantation, respectively. The average estimated C stocks in PMF forest were 81.61, 74.59 and 70.54 t C ha^−1^ for CS_g_, CS_m_ and CS_f_, respectively; and correspondingly 61.72, 54.80 and 54.23 t C ha^−1^ for CAF; and 73.77, 65.00 and 64.81 t C ha^−1^ for CGF. Failing to account for the species- and tissue-specific variations in C concentration introduced a relative error of -1.06% to 13.48% in estimates of biomass C stocks from CS_g_ and CS_f_. However, there was no significant difference (*P* > 0.05) between any estimations of C stock for any given forest (Fig. 5). Across the four forests, there were marginally higher C stocks of CS_g_ than that CS_m_ (*P* = 0.097) and no significant differences of CS_f_ against CS_g_ (*P* = 0.132) and CS_m_ (*P* = 0.877).

## Discussion

Variation among tree species substantially impacted measurements of C concentration. Seventy-two percent of variation in C concentrations was explained by species-specific tissue types and average C concentration differed significantly between tree species, ranging from 42.3% for *A. fortunei* to 47.9% for *C. lanceolata*. This finding is consistent with previous studies^13^. This range is lower than that found for temperate (43.7–55.1%)^8^, boreal (48.4–51.0%)^10^ and subtropical tree species (48.0–57.7%)^14^ in China. It is possible that the relatively low C concentrations in this study may be due to differences in climatic conditions^16, 17^ or tree size^54, 55^. Temperatures in temperate/boreal areas drop below zero and tree species there are known to produce higher molecular weight, C-rich compounds, such as lignin, phenolic acids, suberin and tannins^56^ which leads to higher C concentrations. For tree species growing in similar climatic conditions, tree size is considered to be an important factor. For example, average C concentrations in this study were similar for *C. lanceolata*, about 10.3% lower for *P. massoniana* and 5.4% lower for *C. glauca* compared to the results of Zheng *et al*.^14^. DBH was similar for *C. lanceolata* in this study (15.5 cm) and for Zheng *et al*. (15.1 cm); whereas the DBH range in the present study was greater for *P. massoniana* (5.9–52 cm) and *C. glauca* (6.2–50.9 cm) than found by Zheng *et al*. (8.2–23.7 and 3.2–11.1 cm, respectively).

C concentration differed significantly among stems, bark, branches, leaves, coarse roots and fine roots among tree species. This is in agreement with previous studies showing significant differences in C concentration in stems, branches, leaves and roots of balsam fir^43^ and in stems, new branches, old branches, leaves, coarse roots and fine roots across 10 Chinese temperate tree species^8^. Variation in C concentration among tree tissues was mainly driven by chemical composition. Many studies have shown that stem wood contains two principal chemical compounds: lignin (13.7–35.0% of dry mass) and holocellulose (65.0–75.0% of dry mass)^16^. Branches have similar chemical composition as stem wood, with lignin concentrations of 17.1–27.1% and holocellulose of 26.7–73.0% on dry mass bases^57, 58^. Leaves contain 17.8–35.3% lignin and 36.0–45.0% holocellulose on dry mass bases^57, 59, 60^. The C concentrations we observed were in accordance with the known relative differences in concentration of these compounds^61, 62^, where C concentrations of stems (45.9%) and leaves (45.9%) was higher than in branches (45.1%). Bark has evolved to resist biotic and abiotic stress, leading to similarly high concentrations of C-rich compounds (e.g. extractives, lignin, phenolic acids, suberin and tannins)^54, 63^. These compounds constitute above 40% of dry mass, and C concentration of these compounds is in the range of 59–62% (except for suberin with about 73%)^16^, which can result in higher C concentration in bark. In this study, C concentration was lower in bark (45.0%) than in stems, leaves and branches, while previous studies reported that C concentration was higher in bark than any other tissues^54, 55^. This difference may be due to species growing in different climatic conditions. The C concentrations of coarse (44.0%) and fine (42.0%) roots were lower than all other measured tissues. The concentration of low-C compounds (e.g. starch)^64^ in coarse roots and other low-C compounds in fine roots (e.g. free phenols, bound phenols, lignin phenols and non-structural carbohydrates), compared to other tissues^65, 66^, may explain these differences.

C concentrations were significantly higher for gymnosperm species than angiosperm species (Fig. 3) in this study. Many studies have reported higher C concentrations in gymnosperm species (46.9–57.72%) compared to angiosperm species (43.7–55.1%)^8, 14, 16, 43^. The possible explanation for this is that gymnosperm species generally have higher lignification in tissues^20^, different lignin chemistry^19^ and/or lower concentrations of non-structural carbohydrates^17, 21^. Lignin content can be approximately 30% in gymnosperm stems versus 20% for angiosperms^9^. In addition, Chinese subtropical forests consist of many tree species^35^, making it difficult to measure C concentration for each tree species. Variations in C concentrations among functional types (i.e. gymnosperms and angiosperms) could be a potential alternative to using species-specific C concentrations to estimate forest C stocks at local or large scales.

Estimation of C concentrations was also possible based on characterisation of functional traits. C concentrations of tissues were negatively correlated with LA. The negative relationships between C concentration and LA may be attributable to functional type given the significant phylogenetic signal for LA (Fig. 2). LA is morphological trait known to vary among ecological strategies of tree species^27^. Konôpka *et al*. (2016) noted that leaf size was related to light intensity^26, 67^. Under the same growth condition (e.g. CO_2_ concentration and light intensity), tree species with larger LA exhibit faster photosynthetic rate and higher growth rate than species with smaller LA^24^. In the line with Martin and Thomas^15^, the result of this study showed a negative relationship between C concentrations and RGR but the relationship was not significant. Actually, fast-growing tree species contain lower C-rich compounds (e.g. alkaloids, phenolic glycosides, cyanogenic glycosides), whereas slow-growing species contains higher C concentrations compounds (e.g. lignin, polyphenolic compounds)^8^. Accordingly, LA measurements could provide a reasonable basis for classifying functional types and for estimating the C concentration and C sequestration capacity of tree species.

Failing to account for differences in C concentrations among tree species and tissues introduced significant biases in forest C stock estimates, consistent with previous studies^8, 16^. Estimations of stand C stock using a generic C concentration was 1.91–8.76 t C ha^−1^ (5.45–13.48%) higher than that estimations using direct measurements of species- and tissue-specific C concentrations. Stocks estimation using functional type did not significantly differ from estimations using species- and tissue-specific C concentrations. In CLF, the CS_m_ was just 0.06 t C ha^−1^ lower than CS_f_, as in CAF and CGF, the CS_m_ was 0.57 and 0.19 t C ha^−1^ higher than CS_f_, respectively. In PMF, differences in estimated C stocks were as large as 4.05 t C ha^−1^. This magnitude of error may be due to the higher proportion of angiosperm species (51%) in this forest, which contain lower C concentrations than gymnosperm species. This observation emphasizes the value of basing C stock estimates on functional type when possible. Additional measurements of C concentrations from a greater variety of tree species and tissues will further improve our estimates and is necessary to validate the use of generalised functional types for estimating forest C stocks. Our study demonstrates that profiling forests based on tree functional type provides effective methods for assessing forest C stocks which will continue to improve in accuracy as data on C concentration expands.

## Conclusions

The C concentrations differed significantly among tree species and tissues at our subtropical forest sites. Gymnosperm species exhibited significantly higher C concentrations than angiosperm species. We determined that the use of a generic conversion factor, without considering C concentration differences among tree species and tissues, overestimates C stocks in subtropical forests by 1.91–8.76 t C ha^−1^. Functional type classifications, by leaf morphology and phenological traits, provided comparable estimates of C stocks to those made with species- and tissue-specific C concentrations. Therefore, it is recommended to use functional types in assessing the C sequestration capacity of subtropical forests as it is straightforward and produces comparable results.

## Electronic supplementary material

Supplementary information
