# Supplementary material for: Tree functional types simplify forest carbon stock estimates induced by carbon concentration variations among species in a subtropical area
Source: Sci Rep. 2017 Jul 10;7:4992. doi: 10.1038/s41598-017-05306-z (PMC5504068; doi:10.1038/s41598-017-05306-z)
Supplement: Supplementary file 1 — Supplementary information [file 41598_2017_5306_MOESM1_ESM.doc]

**Tree functional types simplify forest carbon stock estimates induced by carbon concentration variations among species in a subtropical area**

**Huili Wu^1,2^, Wenhua Xiang*^1,2^, Xi Fang^1,2^, Pifeng Lei^1,2^, Shuai Ouyang^1,2^, Xiangwen Deng^1,2^**

^1^ Faculty of Life Science and Technology, Central South University of Forestry and Technology, Changsha, Hunan, 410004, China

^2^ Huitong National Station for Scientific Observation and Research of Chinese Fir Plantation Ecosystems in Hunan Province, Huitong, Hunan 438107, China

*Corresponding author:

Dr. Wenhua Xiang, Faculty of Life Science and Technology, Central South University of Forestry and Technology, No. 498 Southern Shaoshan Road, Changsha 410004, Hunan, China. Email: [xiangwh2005@163.com](mailto:xiangwh2005@163.com); Tel: +86-731-85623350; Fax: +86-731-85623350

**Figure S1.** The relationships of C concentrations in stems, bark, branches, leaves and coarse roots with leaf area (LA) for whole sampling angiosperm trees. The formula Y = a*LA + b was the linear regression equation for the relationship of each tissue C concentration with LA. The *R*^2^ and *P* indicate the linear regression correlation coefficient and significantly value.

**
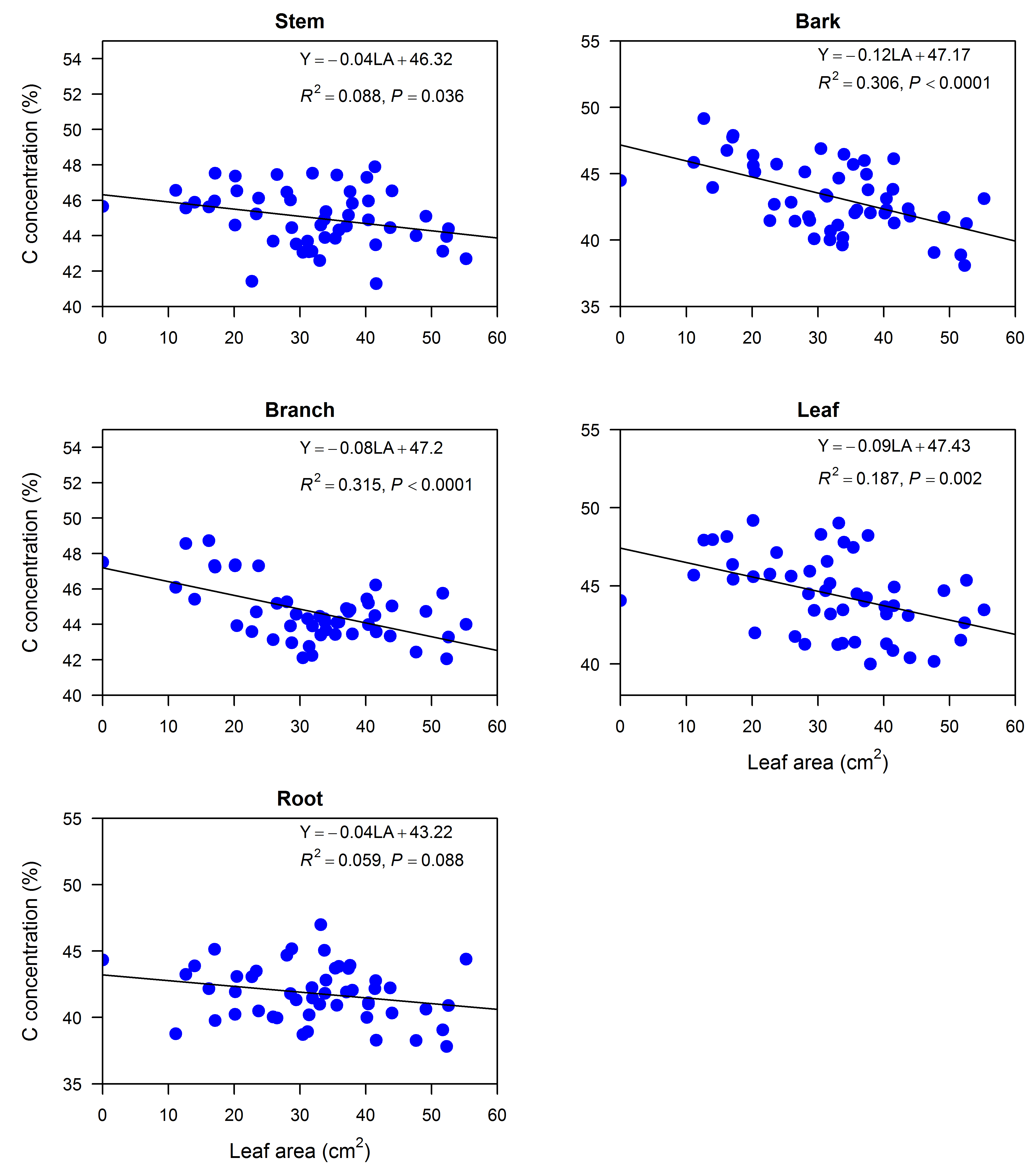
**

**Table S1.** Stand characteristics of the eight forests in which tree C concentrations were sampled.

| Forest types | Number of tree species | Dominant tree species | Density (stems ha^-1^) | DBH (cm) | H (m) | Basal area (m^2^ ha^-1^) |
| --- | --- | --- | --- | --- | --- | --- |
| *Cunninghamia lanceolate* plantation | 1 | Whole stand | 2310 | 15.5 | 15.4 | 40.60 |
|  |  | *Cunninghamia lanceolate* |  |  |  |  |
| *Pinus massoniana*  forest | 16 | Whole stand | 1253 | 12.2(1.6-52.0) | 9.7(1.3-24.0) | 21.87 |
|  |  | Pinus massoniana | 550 | 17.8(2.2-52.0) | 13.6(1.3-24.0) | 16.96 |
| *Alniphyllum fortunei* forest | 16 | Whole stand | 708 | 11.5(0.8-56.0) | 8.5(2.0-26.0) | 14.19 |
|  |  | *Alniphyllum fortunei* | 188 | 11.0(0.8-39.5) | 8.9(3.5-21.5) | 2.41 |
| *Choerospondias axillaris* forest | 11 | Whole stand | 1035 | 10.1(0.4-24.5) | 10.6(1.9-18.0) | 10.15 |
|  |  | *Choerospondias axillaris* | 600 | 9.1(0.4-24.5) | 8.6(1.9-15.9) | 5.77 |
| *Liquidambar formosana* forest | 7 | Whole stand | 682 | 21.6(2.9-58.0) | 16.2(3.5-35.0) | 33.50 |
|  |  | *Liquidambar formosana* | 247 | 19.6(6.9-47.2) | 16.3(7.5-30.2) | 9.11 |
| *Cyclobalanopsis glauca* forest | 4 | Whole stand | 631 | 13.1(2.2-50.9) | 10.1(2.7-23.6) | 12.71 |
|  |  | *Cyclobalanopsis glauca* | 582 | 13.3(2.2-50.9) | 10(2.7-23.6) | 12.03 |
| *Cyclobalanopsis glauca –Litsea rotundifolia* forest | 18 | Whole stand | 503 | 18.1(1.5-80.0) | 12.6(2.0-35.0) | 19.41 |
|  |  | *Litsea rotundifolia* | 74 | 15.2(1.9-45.5) | 10.1(2.0-21.5) | 2.50 |
| *Schima superba*  forest | 13 | Whole stand | 930 | 14.4(1.6-36.5) | 11.0(2.5-26.5) | 22.96 |
|  |  | *Schima superba* | 323 | 11.5(1.6-33.8) | 8.5(2.5-18.5) | 5.90 |

**Table S2.** Effects of species and tissues on C concentrations across all tissues and species. The columns give the degrees of freedom (d.f.), sum of squares (SS), mean of squares (MS), F-values, *P*-values and % deviance explained by the explanatory variables. Significant terms (*P* < 0.0001) are indicated by three asterisks.

| Sources | d.f. | SS | MS | *F* values | *P* values | Deviance  Explained (%) |
| --- | --- | --- | --- | --- | --- | --- |
| Species | 7 | 1698.7 | 242.67 | 75.705 | **<0.0001***** | 35.41 |
| Tissue | 5 | 901.4 | 180.28 | 56.241 | **<0.0001***** | 17.89 |
| Species × tissue | 35 | 866.4 | 24.75 | 7.723 | **<0.0001***** | 18.97 |

**Table S3.** Relationships between C concentrations in each tissue and functional traits. *a* and *b* are the fitted parameters of the formula *y* = *a* + *bx*, where y is C concentration in each tissue and x is the value of functional traits. RMSE is root mean square error; *R*^2^ is the coefficient of determination; n is the number of data. Bold values with asterisk indicate a significant relationship.

| Tissues for C concentrations | n | Traits | *a* | *b* | *F* | RMSE | *R*^2^ | *P* value |
| --- | --- | --- | --- | --- | --- | --- | --- | --- |
| Stem | 8 | LA | 47.63 | -0.079 | 6.089 | 1.496 | 0.504 | **0.049*** |
|  | 8 | SLA | 47.63 | -0.162 | 1.164 | 1.943 | 0.163 | 0.322 |
|  | 7 | MAI | 44.82 | 0.007 | 0.974 | 1.985 | 0.163 | 0.369 |
|  | 7 | RGR | 46.27 | -13.55 | 0.592 | 2.051 | 0.106 | 0.476 |
|  | 8 | WD | 48.89 | -6.200 | 0.968 | 1.971 | 0.139 | 0.363 |
| Bark | 8 | LA | 48.17 | -0.155 | 30.53 | 1.311 | 0.836 | **0.001**** |
|  | 8 | SLA | 46.99 | -0.216 | 0.851 | 3.027 | 0.124 | 0.392 |
|  | 7 | MAI | 44.37 | -0.007 | 0.542 | 2.444 | 0.098 | 0.495 |
|  | 7 | RGR | 43.73 | 0.234 | 0.0001 | 2.573 | 0.000 | 0.992 |
|  | 8 | WD | 53.42 | -17.62 | 5.619 | 2.324 | 0.484 | 0.056 |
| Branch | 8 | LA | 46.71 | -0.072 | 9.803 | 1.081 | 0.620 | **0.020*** |
|  | 8 | SLA | 46.22 | -0.107 | 0.686 | 1.662 | 0.103 | 0.439 |
|  | 7 | MAI | 43.18 | 0.198 | 0.499 | 1.808 | 0.091 | 0.511 |
|  | 7 | RGR | 45.25 | -6.292 | 0.154 | 1.868 | 0.030 | 0.711 |
|  | 8 | WD | 47.67 | -5.290 | 1.044 | 1.619 | 0.148 | 0.346 |
| Leaf | 8 | LA | 48.16 | -0.126 | 14.860 | 1.533 | 0.712 | **0.008**** |
|  | 8 | SLA | 49.16 | -0.319 | 3.180 | 2.310 | 0.346 | 0.125 |
|  | 7 | MAI | 45.83 | -0.011 | 2.182 | 1.966 | 0.304 | 0.200 |
|  | 7 | RGR | 45.42 | -10.470 | 0.283 | 2.292 | 0.054 | 0.618 |
|  | 8 | WD | 51.67 | -12.26 | 2.569 | 2.391 | 0.300 | 0.160 |
| Coarse root | 8 | LA | 46.34 | -0.118 | 15.19 | 1.418 | 0.717 | **0.008**** |
|  | 8 | SLA | 45.92 | -0.207 | 1.201 | 2.433 | 0.167 | 0.315 |
|  | 7 | MAI | 42.939 | -0.001 | 0.007 | 1.998 | 0.001 | 0.936 |
|  | 7 | RGR | 42.953 | -1.250 | 0.005 | 1.998 | 0.001 | 0.945 |
|  | 8 | WD | 50.89 | -14.518 | 5.612 | 1.916 | 0.483 | 0.056 |
| Fine root | 8 | LA | 43.24 | -0.091 | 2.145 | 2.906 | 0.263 | 0.193 |
|  | 8 | SLA | 43.90 | -0.244 | 1.006 | 3.134 | 0.144 | 0.355 |
|  | 7 | MAI | 40.59 | -0.004 | 0.184 | 2.399 | 0.035 | 0.686 |
|  | 7 | RGR | 40.15 | 1.492 | 0.005 | 2.441 | 0.001 | 0.946 |
|  | 8 | WD | 44.98 | -7.699 | 0.552 | 3.241 | 0.084 | 0.486 |
